# Review of Plasma Processing for Polymers and Bio-Materials Using a Commercial Frequency (50/60 Hz)-Generated Discharge

**DOI:** 10.3390/polym15132850

**Published:** 2023-06-28

**Authors:** Hong Tak Kim, Cheol Min Jung, Se Hyun Kim, Sung-Youp Lee

**Affiliations:** 1 Department of Physics, Kyungpook National University, Daegu 41566, Republic of Korea; zam89blue@gmail.com; 2 Division of Chemical Engineering, Konkuk University, Seoul 05029, Republic of Korea; daesil159@gmail.com

**Keywords:** dielectric barrier discharge (DBD), commercial frequency, 50/60 Hz, plasma, PECVD, polymer, sputtering, surface modification

## Abstract

This manuscript introduces the properties and diverse applications of plasma generated using commercial frequencies of 50/60 Hz. Commercial frequency (CF) derived plasma exhibits characteristics similar to DC discharge but with an electrical polarity and a non-continuous discharge. Due to the low-frequency nature, the reactor configurations usually are capacitively coupled plasma type. The advantages of this method include its simple power structure, low-reaction temperature, and low substrate damage. The electrical polarity can prevent charge buildup on the substrates and deposited films, thereby reducing substrate damage. The simple, low-cost, and easy-to-operate power structure makes it suitable for laboratory-scale usage. Additionally, the various applications, including plasma-enhanced vapor deposition, sputtering, dielectric barrier discharge, and surface modification, and their outcomes in the CF-derived plasma processes are summarized. The conclusion drawn is that the CF-derived plasma process is useful for laboratory-scale utilization due to its simplicity, and the results of the plasma process are also outstanding.

## 1. Introduction

**Figure 1:**
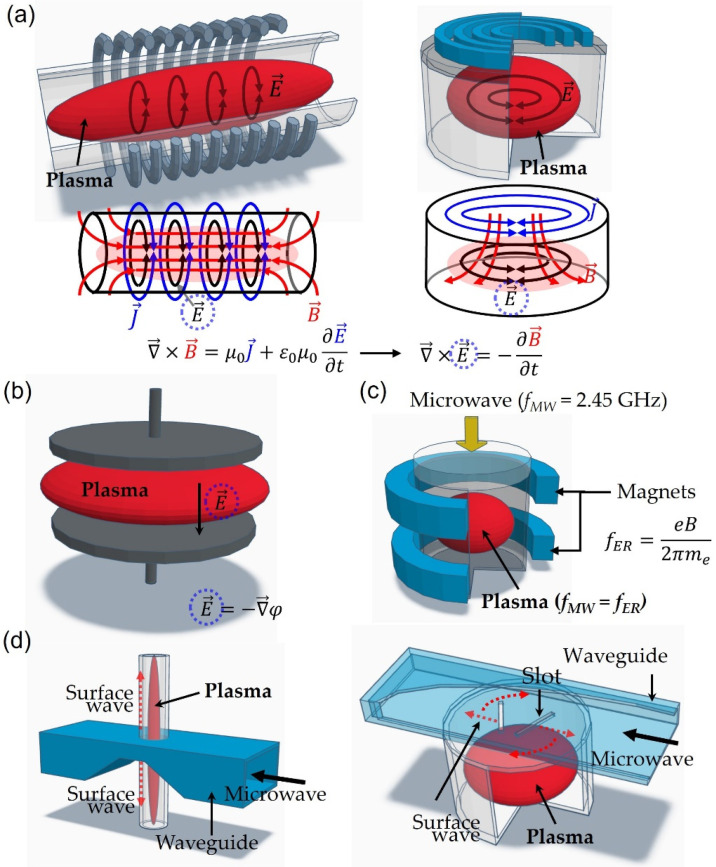
Schematic diagrams for plasma generation with different configurations: (**a**) inductively coupled plasma (ICP): Maxwell equations related to the generation of ICP and the schematic diagram for the applied current ($\vec{J}$: current density, solid blue line), induced magnetic field ($\vec{B}$, solid red line), and electric field (solid black line) in different ICP configurations; (**b**) capacitively coupled plasma ($\vec{E}$: electric field; φ: electric potential); (**c**) microwave plasma: electron resonant plasma (*f_MW_*: microwave frequency; *f_ER_*: electron resonance frequency); (**d**) microwave plasma: surface-wave sustained plasma.

Plasma is a partially ionized gas consisting of equal numbers of positively charged ions, negatively charged electrons, and neutral particles. There are two main types of plasma: thermal plasma and non-thermal plasma. Thermal plasma is in thermal equilibrium, meaning that the electron and ion temperatures are the same. Thermal plasma is commonly used in high-temperature industrial applications such as welding, cutting, and melting materials [1]. On the other hand, non-thermal plasma is not in thermal equilibrium, meaning that the electron temperature is much higher than the ion temperature. Thus, the gas temperature of non-thermal plasma is low and is usually used in material processing [2,3,4]. In addition, non-thermal plasma is appealing because it can activate chemical and physical reactions at low temperatures compared to non-plasma methods [2,3,4]. Non-thermal plasma processes are plasma-based material processing techniques. These can be used in deposition techniques such as plasma-enhanced vapor deposition (PECVD) [5,6] and sputtering [7,8,9,10], etching (RIE) [11,12], surface modification [13,14], and cleaning processes [15,16]. As a result, the plasma method has been extensively employed in the micro/nanofabrication of electronic devices, surface cleaning and modification of various substrates, trash disintegration, and food applications [17,18]. In addition, a plasma discharge is a strong ultraviolet (UV) source, and UV alone can be used in various applications, such as the photolithography process and surface modification [19,20]. From the point of view of the generation method, the plasma sources can be divided into capacitively coupled plasma (CCP), inductively coupled plasma (ICP), and microwave plasma (MWP), as shown in Figure 1. The most prevalent method for generating plasma is CCP, which involves the use of two metal electrodes separated by a discharge gap. The power is applied to the electrodes and the plasma is directly generated using an electric field, as depicted in Figure 1b. In the case of ICP, an alternating-current (AC) power, typically at a frequency of 13.56 MHz, is applied to an external coil or antenna. The time-varying current flowing through the coil generates a magnetic field around the external coil (or antenna). Then, the electric field is induced by the magnetic field, as shown in Figure 1a. Finally, the plasma is generated using the induced electric field formed inside the reactor.

The MWP can be classified as electron cyclotron plasma and surface-wave sustained plasma [2,3,21,22,23,24]. In electron cyclotron plasma, all free electrons rotate with the same frequency (electron cyclotron frequency: ECF) in a strong magnetic field. In this situation, the applied microwave frequency (MWF) matches the ECF, and the resonance occurs between the MWF and ECF. The resonating effect heats many electrons and generates a plasma discharge [21], as shown in Figure 1c. In surface-wave sustained plasma, the discharge is generated via the propagation of electromagnetic surface waves [22,23,24], as shown in Figure 1d. Table 1 shows a summary of plasma parameters at different plasma-generating methods. Similarly, in terms of power frequency, the plasma sources can be classified as direct current (DC), commercial frequency (CF, 50/60 Hz), mid-frequency (MF, ~kHz), radio frequency (RF, 13.56 MHz), and microwave frequency (MWF, 2.45 GHz). In most cases, a low-frequency, including 50/60 Hz, has been applied to the CCP method instead of the ICP method. The reason for this is that the slowly varying input current creates a magnetic field that makes it challenging to form an induced electric field sufficient to cause a discharge. Commercial-frequency (CF, 50/60 Hz) derived plasma is similar to DC discharge with polarity, and exhibits different properties compared to the high-frequency derived plasma. In addition, a CF power source has a very simple and easily made design, which is inexpensive to build. However, CF-derived plasma processes have not been widely used despite their many advantages, and studies have been performed by very few groups and focused on film depositions using plasma-enhanced chemical vapor deposition [25,26,27,28,29,30,31,32,33,34,35,36,37,38,39,40]. Recently, CF-derived discharge has been applied not only to low-pressure discharge but also to atmospheric discharge. This review focuses on the design of CF power sources, the properties of CF-derived plasma, and applications of CF-derived plasma processes.

## 2. Plasma Properties Generated Using a Commercial-Frequency (50/60 Hz) Power Source

### 2.1. Nonthermal Plasma

Typical classifications of plasma identify two main categories as equilibrium and non-equilibrium plasma [1,2,3,4]. In an equilibrium plasma, the temperatures of all species (electrons, ions, and neutral gases) in plasma are almost same. Thus, the gas temperature is high, which is called thermal plasma. The main heating mechanism is joule heating and thermal ionization. A representative example of thermal plasma is arc plasma. The main disadvantages of thermal plasma are high gas temperatures and low excitation selectivity. Thus, the applications of thermal plasma are limited to cutting, welding, and decomposition of various materials. In non-equilibrium plasma, the temperature of electrons is much higher than that of ions and neutral gases. Although the electron temperature is high, the gas temperature is low because neutral gases make up the majority of plasma. Thus, non-equilibrium plasma is called non-thermal plasma, and the electron impact process plays a crucial role in determining the plasma properties. Examples of non-thermal plasma are low-pressure glow discharge (CCP, ICP, and microwave plasma), atmospheric DBD plasma, and so on. Due to the low gas temperature and high excitation selectivity of non-thermal plasma, the plasma has been applied in many fields. The processing plasma can be classified into corona, the glow, and arc discharge according to the discharge current. The discharge current is in the range of 10^−7^–10^−5^ A for corona discharge, 10^−5^–1 A for glow discharge, and more than 1 A for arc discharge [45]. Thus, corona and glow discharge are non-thermal plasma, and arc plasma is thermal plasma. The properties of thermal and non-thermal plasma are summarized in Table 2. In addition, a plasma simulation is also a good tool for predicting discharge characteristics. There are a few plasma simulation studies that have used commercial frequencies; so, we briefly introduce plasma simulation here [46,47,48]. Plasma simulations can be divided into particle-in-cell (PIC), kinetic simulation and fluid simulation. PIC simulations do not require the assumption required in fluid simulation and has the advantage of high accuracy via directly calculating the motion of charged particles. However, if enough particles are not introduced, there is a statistical error, and the introduction of many particles requires a large number of calculations; so, there is a limit to the calculation speed. The Boltzmann equation solver, which uses the Eulerian scheme, while calculating the velocity space, has high accuracy. Still, it is difficult to calculate this using multi-dimensional calculations that deal with speeds and spaces in two or more directions. For this reason, the Boltzmann equation is solved in many cases due to collisions in 0 and 1 dimensions. Currently, the most used code uses a hybrid method that combines Monte Carlo collision calculation techniques and 0-dimensional Boltzmann solvers based on fluid techniques to easily handle various species [47]. These two codes improve the accuracy of ionization and the generation of excitation species caused by electrons and ions colliding with neutral gases. In the case of high pressure and various gas types, most researchers use fluid simulation.

### 2.2. Design and Construction of CF power Source

**Figure 2:**
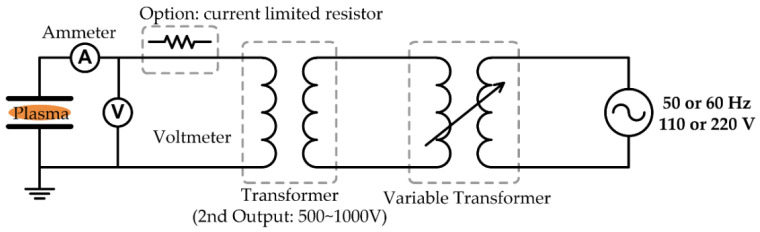
Schematic diagram of a low-frequency power source for plasma discharge generation.

A CF (50/60 Hz) power simply consists of a transformer and a voltage controller. Usually, the variable transformer, called VARIAC or power-stat, is used as the voltage controller, and the output voltage is boosted using a transformer with an appropriate step-up ratio. A secondary output voltage of the transformer is about 500–1000 V for low-pressure conditions, and several kV for high-pressure conditions, including atmospheric discharge. The voltmeter and ammeter are needed for the voltage and current measurements during plasma discharge. The CF power source does not necessitate a complicated power conversion system, such as a matching network and frequency generation components. As a result, the power structure is straightforward, cost-effective, and easy to use in a laboratory. At high-pressure and high-voltage discharge conditions, such as atmospheric conditions, the plasma tends to shift from glow discharge to arc spark due to a slow change in voltage polarity [25,26,51,52,53]. Thus, the power is often applied to the electrode through a suitable resistor to limit the discharge current. Moreover, it is recommended to use a suitable fuse to block the overcurrent for safety. Figure 2 shows the schematic diagram of a CF (50/60 Hz) power source.

### 2.3. Plasma Characterizations and Properties Generated Using CF power

**Figure 3:**
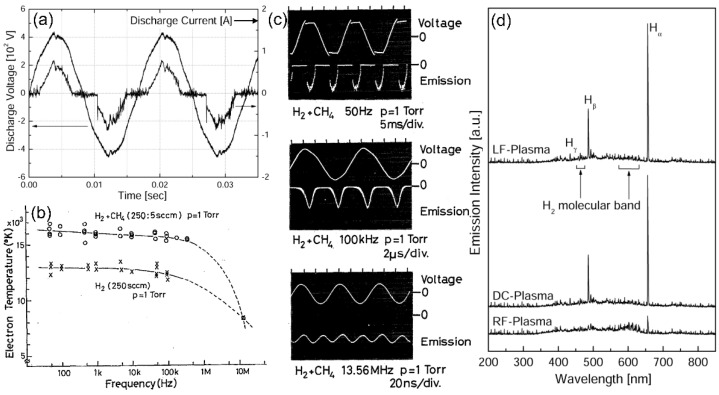
Properties of plasma discharge generated using a commercial frequency (CF, 50/60 Hz) power source and comparison of results for DC- and RF-derived plasma: (**a**) time-resolved applied voltage and discharge current for Ar plasma discharge [29], (**b**) change in the electron temperature in H_2_ and H_2_-CH_4_ plasma as a function of plasma power frequency (○: H_2_ + CH_4_, ×: H_2_) [28], (**c**) oscillograms of optical emission intensities and applied voltage at different power frequencies [28], and (**d**) optical emission spectra of H_2_ plasma generated using DC, CF, and RF power sources [29]. ((**a**,**d**) reprinted with the permission from Ref. [29]. (2003, KPS). (**b**,**c**) reprinted from Ref. [28]. (1991, AIP Publishing).).

The CF-derived plasma is a short-lived discrete discharge similar to the pulsed DC discharge with changing polarity. Figure 3a shows the oscillogram of current and voltage for a CF-derived H_2_ discharge. The phase shift between applied voltage and discharge current was not observed in the CF-derived plasma, implying that the CF-derived plasma had resistive properties [27]. Kim et al. investigated plasma parameters of CF-derived discharge using hydrogen, argon, and hydrogen-methane gas [27,29,30,35]. The plasma was generated using the CCP type (electrode diameter: 10 cm, electrode gap: 3–5 cm, pressure: 0.4–1.2 Torr, Power: 10–50 W). The plasma parameters were acquired using a Langmuir probe method [2,3,4,35,45], and the electron energy distribution is assumed to follow the Maxwell distribution. In the plasma, the electron current (*I_e_*) on the probe as a function of applied voltage (*V*) is given by:

(1) $$I_{e}\left(V\right)=j_{es}A_{p}exp\left(-\frac{e(V_{p}-V)}{kT_{e}}\right)$$

Where, *j_es_* is the saturation current density, *A_p_* is the surface area of the probe tip, *V_p_* is the plasma potential, *k* is the Boltzmann constant, and *T_e_* is the electron temperature (unit: eV). After taking the natural log in Equation (1) and plotting ln(*I_e_*) with respect to *V*, the inverse of the slope for the graph is *T_e_*. Using the values of *T_e_*, *A_p_*, and *I_es_*, *n_e_* is given by:

(2) $$n_{e}=3.78\times 10^{11}\frac{I_{es}}{A_{p}T_{e}^{1/2}}$$

Where, the unit of *T_e_* is the electron volt (eV). The measured values of *T_e_* and *n_e_* for the hydrogen, argon, and hydrogen-methane plasma were 1.5–3.3 eV and n_e_ = ~10^8^ cm^−3^. During Ar discharge, the increase in pressure from 400 to 1200 mTorr caused the decrease in *T_e_* (3.3 eV → 2.6 eV), while *T_e_* seldom changed at about 3.2 eV, according to the discharge current (pressure: 600 mTorr) [27,30]. In H_2_-CH_4_ plasma, *T_e_* increased from 2.4 eV to 3.2 eV with the increase in power from 10 W to 45 W [30,35]. Lau et al. measured the discharge parameters of CF-derived Ar plasma using a Langmuir probe [54]. The plasma was generated using a CCP type (electrode diameter: 4.5 cm, electrode gap: 3 cm, power: 5–35 W, pressure: 0.9 Torr). The measured *T_e_* and *n_e_* were in the range of 2–3 eV and ~10^8^ cm^−3^, respectively. These parameters were similar values compared to those of the high-frequency plasma method. Shimozuma et al. obtained the *T_e_* in H_2_ and H_2_-CH_4_ plasma as a function of discharge power frequency, as shown in Figure 3b. The plasma was generated in a CCP type (electrode diameter: 12 cm, gap: 2 cm, pressure: 1.0–1.5 Torr), and *T_e_* was evaluated using the relative intensity method, which is given by [27,28,45,55]:

(3) $$\frac{I_{ij}}{I_{kl}}=\frac{\lambda_{kl}A_{ij}g_{i}}{\lambda_{ij}A_{kl}g_{k}}\exp\left(-\frac{E_{i}-E_{k}}{k_{B}T_{e}}\right)$$

where, *I_ij_* and *I_kl_* are the spectral intensity, *λ_ij_* and *λ_kl_* are the wavelengths, *A_ij_* and *A_kl_* are the transition probabilities, *g_i_* and *g_k_* are the statistical weights, and *k*_B_ is the Boltzmann constant. *T_e_* values at 1 kHz and 13.56 MHz were 16,000 K (1.4 eV) and 8200 K (0.71 eV), respectively. The discharge mode also changed from discontinuous to continuous at a power frequency of about 200 kHz, as shown in Figure 3c. In addition, the intensity ratio of the excited molecular hydrogen (H_2_*) to H_α_ and the ratio of H_2_* to H_ß_ in 13.56 MHz-generated plasma was much larger than that in CF-derived discharge [28]. The authors concluded that the electron temperature (*T_e_*) in CF-derived plasma is higher than it is in RF-derived plasma [28]. However, the relationship between power frequency and electron temperature cannot be easily generalized without considering the specific system conditions, including the geometric configuration of electrodes, discharge gas type, discharge pressure, discharge voltage, and so on. Kim et al. studied the molecular excitations in H_2_ plasma generated at different power frequencies, as shown in Figure 3d [29]. The intensity of molecular excitations in DC- and CF-generated plasma was less than that in RF-generated plasma. This meant that the energy consumption for molecular excitations in RF-generated discharge was much higher than that at DC- and CF-generated plasma. Thus, the main portion of input energy of the molecular gas discharge is consumed for the excitation of molecular vibration, and the energy loss is larger than that of high-frequency discharge [30,56,57]. This difference can be explained as a plasma heating mechanism. In non-thermal plasma, the electron impact processes play a crucial role in the energy transfer, and there are two modes to heat plasma: ohmic heating and stochastic heating. Electrons absorb energy from the applied electric field, and electron-particle collisions produce ohmic heating in bulk plasma. On the other hand, the momentum transfer due to sheath oscillation leads to stochastic heating at the sheath edge [2,3]. Generally, electron heating at a low frequency is dominated by the direct absorption of energy from the electric field. In contrast, the heating mechanism shifts towards stochastic heating processes at a high frequency.

In summary, CF-derived plasma was a discontinuously discharged, while DC- and RF-derived plasma were continuously discharged. The electron temperature and density were in the range of a few eV and 10^8^–10^9^ cm^−3^, respectively, and these values are similar to those of other CCP plasma. The direct comparison of plasma parameters for discontinuous and continuous discharges required some attention. In the case of continuous discharge, the mean value could express plasma characteristics because the plasma maintained the glow discharge after discharge build-up. In periodically repeating plasma, the build-up from Townsend to glow discharge was repeated according to power frequency, and this implied the plasma characteristics periodically changed in one discharge period. Ohmic heating was the main heating mechanism in DC- and CF-derived plasma, and the heating mechanism shifted to stochastic heating in RF-derived plasma. Considering plasma processing, CF- and RF-derived plasmas could be applied to the processing of most materials, such as metals and dielectrics. In contrast, they could be applied only to metal deposition and surface treatments for DC plasma. CF-derived plasma has a slower processing speed than DC- and RF-derived discharges do because of the short plasma duration time. Table 3 summarizes the comparisons of DC-, CF-, and RF-derived plasma characteristics in CCP-type discharges. In addition, it is also important to understand the difference between CF- and kHz-derived plasma. Both are discrete discharges, and the difference between CF- and kHz-derived plasmas is the degree of pre-ionization in the off-state of discharges. Pre-ionization refers to the charges that remain in the off-state. As the power frequency in the discontinuous discharge mode increased, the effect of the pre-ionized charge also played an important role in generating plasma, which mainly affected the discharge firing voltage and discharge lag time [58,59,60]. Thus, CF plasma has a higher discharge firing voltage than kHz-derived plasma does.

## 3. Plasma Processes Using CF-Derived Discharge

### 3.1. Film Depositions on Polymer and Polymeric Materials Using CF-Derived Discharge

Chemical vapor deposition (CVD) is a common thin film growth method based on a chemical reaction on a substrate surface [61]. Typical CVD uses thermal energy to drive the chemical reaction and usually requires high temperatures. PECVD is a kind of CVD that combines thermal and plasma-derived chemical reactions [2,3,4]. The plasma is used to dissociate reactant gases that then recombine on the sample surface into the desired material. Chemical reactions can activate the usage of plasma, and the reaction temperature can be drastically lowered. Thin film formation using the commercial frequency (CF) PECVD technique was first developed by Shimozuma et al. [31]. They grew Si_3_N_4_, SiO_2_, and a-Si:H films on GaAs and Si substrates using CF-PECVD [31,32,33,34]. The as-deposited films showed good properties, and the deposition temperature was relatively low, below 200 °C compared to another plasma CVD method. Kim et al. also reported the growth of a-C:H films on glass substrates at room temperature and acquired crack-free films [35]. In addition, wear-resistant depositions, including TiN and TiC films, were performed at relatively low temperatures (350–500 °C), and the resulting films exhibited good properties [36,37,38,39,40]. However, the film depositions using CF-PECVD were not applied to polymer substrates. The film depositions on polymer substrates were performed using the CF sputtering technique [62,63,64,65].

**Figure 4:**
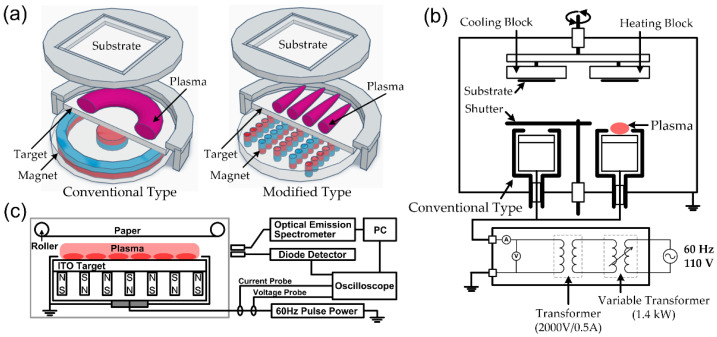
Magnetron sputtering system for transparent conductive thin film deposition using a commercial-frequency (CF) power source: (**a**) comparison between conventional sputtering gun (**left**) and modified sputtering gun with parallel magnet array (**right**), (**b**) ITO and IZO film depositions on glass and polymer substrates using CF (60 Hz) sputtering [63,64], and (**c**) ITO film depositions on paper substrates using CF (60 Hz) DC-pulsed sputtering [65]. ((**c**) reprinted with permission from Ref. [65]. 2021, Elsevier).

Magnetron sputtering deposition is a kind of physical vapor deposition. This technique uses a confined plasma to sputter vaporized atoms from the precursor target, and the vaporized atoms form thin films on the surface of the samples [2,3,4,7,8,9,10]. The samples can be held at RT or heated at the desired temperature during film depositions. Moreover, oxide or nitride materials can be obtained using a reactive sputtering technique. This technique involves the sputtered atoms reacting with gases, such as oxygen and nitrogen, upon condensation on the sample. The film deposition on polymer substrates has many limitations due to low heat-deflection temperature and the weak surface of polymers. Thus, polymer substrates can be easily damaged under the deposition process, and this can affect the characteristics of as-deposited films on polymer and polymer-like materials. In this respect, CF-generated magnetron sputtering can be considered one of the growth techniques on polymer and polymer-like substrates. Jung et al. and Kim et al. reported the deposition of transparent conductive oxides on various polymers using the same CF-sputtering system [62,63], which was a conventional type sputtering gun, as shown in Figure 4a. Jung et al. studied the effects of film thickness and growth temperature for ITO film depositions on polyether-sulfone (PES) substrate [62]. A distance of 10 cm was kept between the ITO target (In_2_O_3_:SnO_2_ = 9:1 wt%, 3 inches) and the substrate, and the applied voltage was maintained at 280 V. Ar was used as the sputtering gas (pressure: 2.2 mTorr, flow rate: 30 sccm), and the growth rate of the film was about 7 nm/min. As the growth temperature increased from RT to 140 °C, the sheet resistance changed from 69 to 151 Ω/sq., the roughness decreased from 1.1 to 0.8 nm, and the optical band gap increased from 3.61 to 3.83 eV. Due to the film thickness change from 134 to 237 nm, the surface roughness increased from 1.2 to 2.2 nm, and the sheet resistance decreased from 316 to 56 Ω/sq., whereas other properties showed a little variation. All films showed good transparency (~85%) in the visible wavelength region and an amorphous phase. Kim et al. reported the growth of indium zinc oxide (IZO) film on various polymers such as PES, PET (polyethylene terephthalate), and PC (polycarbonate) [63]. The sputtering system was the same as mentioned above [62], and the difference was the target material, which was composed of In_2_O_3_ and ZnO (9:1 wt%). The Ar gas flow rate was set to 30 sccm, and the working pressure was maintained at 1.9 mTorr to generate plasma. The plasma was generated at different voltages between 300 V and 320 V, and the polymer substrates were kept at RT during IZO growth. With an increase in the applied voltage, the growth rate of IZO films increased from 4.5 nm/min to 6.5 nm/min. The resulting IZO films were amorphous with an average transmittance of approximately 84% and had a very smooth surface with a surface roughness (*R*_rms_) of about 2 nm. These excellent properties demonstrate that the CF technique is suitable for polymer substrates. Another application of CF-derived plasma is film deposition on a paper substrate. Kim et al. reported an ITO film deposition on a paper sheet using the CF DC-pulsed magnetron sputtering technique [65]. They used a different concept to design the sputtering gun, as shown in Figure 4a; Figure 4c represents the schematic diagram of the CF DC-pulsed sputtering for ITO film deposition on paper sheets. They modified the magnetic array inside a magnetron gun to acquire a magnetic field parallel to the substrate. The size of the ITO target (In_2_O_3_:SnO_2_ = 9:1 wt%) was 416 mm × 298 mm, and its distance from the substrate was 60 mm. Ar-O_2_ mixing gas (Ar:O_2_ = 98:2, 1 sccm) and pure Ar gas (185 sccm) were utilized as sputtering gases (working pressure: 3 mTorr).

The plasma was generated using DC-pulsed power with a negative square pulse type and a frequency of 60 Hz. The film growth was carried out on paper sheets at a power of 93 W (292 V, 32 mA), with a growth rate of 40 nm/min. The ITO films that were deposited exhibited desirable characteristics, including a high level of transmittance (>85%), a good sheet resistance (40 Ω/sq.), a hydrophobic surface with a water droplet contact angle of 116°, and a cubic crystal structure with a grain size of 20.5 nm. Most of all, the fabric structure of the paper after ITO film depositions seldom changed compared to the original paper, and the ITO-deposited sheet did not bend due to the residual stress of ITO films. Compared to DC and RF methods, CF-derived plasma sputtering could reduce the rapid heating of samples due to the incidence of high-energy particles. And this method could prevent sample damage from occurring due to the accumulation of charged particles. Because CF-derived plasma continuously changes the polarity of the discharge [65,66]. On the other hand, the plasma processing time was longer than that of continuous discharges. These results [62,63,64,65] indicate that the CF-derived plasma sputtering method is effective for film depositions on soft substrates, including papers, polymers, and fibers.

### 3.2. Surface Modifications of Polymer and Polymer-like Materials Using CF-Derived Discharge

**Figure 5:**
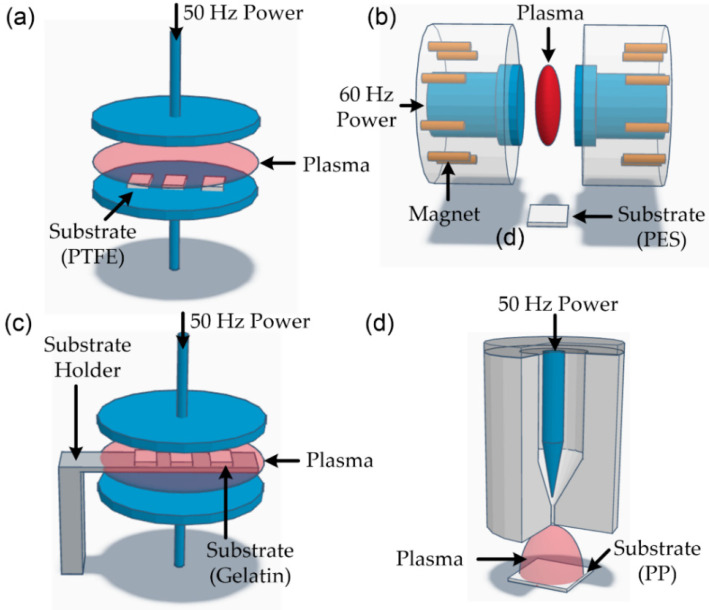
Polymer surface treatments using commercial-frequency (50/60 Hz) plasma source: (**a**) simple diode configuration for surface modification of polytetrafluoroethylene [54], (**b**) magnetic enhanced diode-electrode arrangement for surface treatment of polyether-sulfone film [70], (**c**) simple diode configuration for plasma generation (the substrate holder was positioned at the center of two electrodes) [71,73,74], (**d**) plasma torch configuration [80].

CF-derived plasma techniques have been widely explored for surface modification of various materials. Surface modification of polymers is especially crucial in achieving desirable surface properties such as adhesion, wettability, and biocompatibility [13,14,41,66,67,68,69]. To this end, Lau and colleagues conducted a study on the surface modification of polytetrafluoroethylene (PTFE) films using Ar plasma generated using CF power at a frequency of 50 Hz [54]. The discharge reactor was a traditional diode configuration (see Figure 5a), and Ar plasma (applied voltage: 240 V, pressure: 120 Pa/0.9 Torr) was used to modify the surface of PTFE films. The measured electron temperature and density were 2–3 eV and ~10^8^ cm^−3^, respectively. After plasma exposure, the contact angle for the water was changed from 114° to 91° with varying applied power. They claimed that wettability increased from incorporating oxygen-containing functional groups on the treated surface and the concomitant reduction in fluorine elements. Bhak et al. performed the surface treatment of PES using a magnetized plasma and a CF (60 Hz) power source [70]. The traditional diode-type configuration was used to generate plasma discharge. Each electrode had an array of Nd-Fe-B permanent magnets, similar to a magnetic mirror, as shown in Figure 5b. After subjecting the water droplets to Ar plasma treatment (pressure: 800 mTorr, power: 20 W, treatment time: 0–20 min), the contact angle decreased from 80° to 30°, while the surface roughness of the films remained unchanged (~3.0 nm). These results indicate that the magnetized plasma did not cause any damage to the surface of the films, and it was attributed to the magnetic field’s ability to trap energetic particles.

The application of plasma surface treatment has proven to be advantageous in the field of biomaterials, specifically for gelatin. Gelatin is a transparent and colorless protein commonly derived from the partial hydrolysis of native collagen. Gelatin is a biocompatible, biodegradable, non-immunogenic, and non-antigenic material [71,72]. Gelatin has been widely used as a gelling substance in food, medications, drug capsules, photographic films, and cosmetics. Prasertsung et al. reported the surface modification of cross-linked gelatin films using oxygen, nitrogen, and air glow discharge generated using a 50 Hz power source [71,73,74], and the configuration of the discharge cell was a traditional diode structure, as shown in Figure 5c. After the O_2_, N_2_, and air plasma treatment, in this order (pressure: 1 mbar, treatment time: 1–13 s), the contact angle for a water droplet dramatically decreased to about 23° compared to untreated films (water contact angle: 87°). For all cases, the surface energy of the gelatin films rose progressively up to a plasma exposure duration of 15 s, after which the energy values stabilized. Notably, the polar components of the surface energy increased, while the dispersive components decreased, indicating the emergence of polar functional groups on the surface of the gelatin films as a result of the plasma treatments. The plasma, which was oxygen-containing, facilitated the formation of polar functional groups, including C=O and O-C=O groups, on the polymer substrates’ surface, resulting in the surface’s hydrophilic properties [75] and N_2_ plasma treatment played a similar role on the surface of gelatin films. Prasertsung et al. also studied the attachment and growth behavior of mouse fibroblasts (L239) and rat bone marrow-derived mesenchymal stem cells (MSCs) on nitrogen plasma-treated gelatin films using the same plasma treatment system. The suitable water contact angle and O/N ratio of nitrogen plasma-treated gelatin film for best L929 and MSC attachment were 27°–32° and 1.4, respectively [71,74].

Recently, atmospheric plasma was generated using a CF (50/60 Hz) power source, and the plasma was used as the surface treatment method. Atmospheric plasma can be widely used for surface modification because of the eco-friendly properties of the plasma and inexpensive methods compared to vacuum plasma treatments. Usually, nonthermal atmospheric plasma is generated using high-frequency power sources of several kHz or more [76,77,78,79]. Choi et al. developed the nonthermal atmospheric pressure plasma torch, operated by a 60 Hz power source with a neon transformer (output voltage: 4 kV, output current: 120 mA) [80]. The cold plasma torch (N_2_ flow rate: 40 slm, applied voltage: ~2 kV), as shown in Figure 5d, was applied to polypropylene polymer substrates to enhance the bonding strength, which is evaluated by the lap shear strength. The plasma torch showed a low gas temperature below 60 °C, and the mainly observed excitation emissions were N_2_* (C^3^Π_u_ → B^3^Π_g_) at a wavelength of 337.1 nm, N_2_* (B^3^Π_u_^+^ → A^3^Σ_u_^+^) at a wavelength of 715.3 nm, and N_2_^+^ (B^2^Σ_u_^+^ → X^2^Σ_g_^+^) at a wavelength of 391.4 nm. As a result, the maximum bond strength of about 10.5 MPa was obtained at the optimal condition, and the value is about 60 times higher than that of plasma-untreated samples. According to the authors, the use of excited species such as N_2_* and N_2_^+^ can create polymer-excited states, which increases the population of oxygen and nitrogen atoms on the polymer surface and subsequently improves bonding strength.

**Figure 6:**
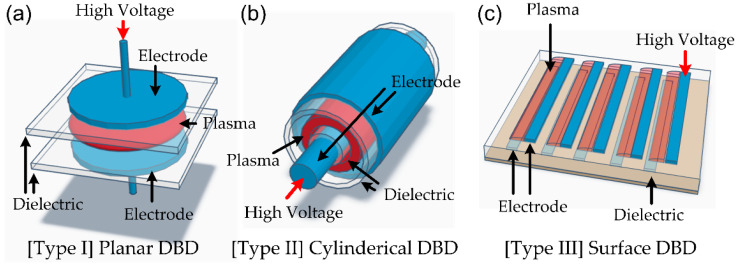
Schematic diagram of typical dielectric barrier discharge (DBD) configurations: (**a**) planar-type DBD source, (**b**) cylindrical type DBD source, and (**c**) surface-discharge type DBD sources.

Joshi et al. employed a surface treatment technique using a DBD generated using a 50 Hz power source [81]. Two rectangular copper electrodes (size: 5 cm × 3.5 cm × 1 cm, gap distance: 3.5 mm) and the lower electrode were covered by a polycarbonate (PC, size: 10 cm × 8 cm × 0.2 cm) plate, resulting in the type of DBD (see Type 2 in Figure 6). The plasma discharge was generated via applying a voltage of 13 kV, and a mixture of air-argon gas (Ar flow rate: 2slm) was used as the treatment gas. Following plasma treatment lasting between 5 and 60 s, the water contact angle on untreated PP films was 93.7° (with a surface energy of 36.7 mJ/m^2^). However, after a treatment time of just 5 s, the contact angle decreased significantly to 67.3° (with a surface energy of 41.6 mJ/m^2^), and no further change was observed with increased treatment time.

### 3.3. In-Package Cold Plasma Treatment on Organic Materials Using CF-Derived Plasma

In-package plasma is a type of plasma technology that involves using a plasma source integrated into a packaging material or product [82,83,84,85,86,87,88]. This allows for the inactivation of microorganisms and other pathogens on the product’s surface without additional processing steps. The plasma source used in in-package plasma systems is typically a non-thermal plasma, which means that it operates at relatively low temperatures compared to thermal plasmas. Non-thermal plasma sources can generate a variety of reactive species, such as ozone, hydrogen peroxide, and other radicals, which can effectively inactivate microorganisms on surfaces. The generation method for in-package plasma is usually a dielectric barrier discharge (DBD). The DBD is a form of CCP, and the plasma is generated between two electrodes separated by an insulating (or dielectric) barrier, as shown in Figure 6. The dielectric barrier can stop excessive currents and prevent arc (or spark) formation. The DBD has been widely applied in various fields: ozone generation, ultraviolet (UV) and excimer light sources, polymer surface modification, bio/medical applications, air pollutant cleaning, etc. [3,41,67,68,69,89,90,91,92,93,94,95,96]. Usually, mid-frequency power (typically 10–30 kHz) is used to generate DBD, and DC discharge cannot be applied due to the existence of the dielectric barrier [3,41,67,93]. CF power is also applied to generate DBD plasma, which does not require an impedance-matching network. This simplicity and affordability make DBD systems more practical for industrial applications. DBD devices can be constructed in various configurations, and planar, cylindrical, and surface-discharge type configurations (Figure 6) are widely used.

**Figure 7:**
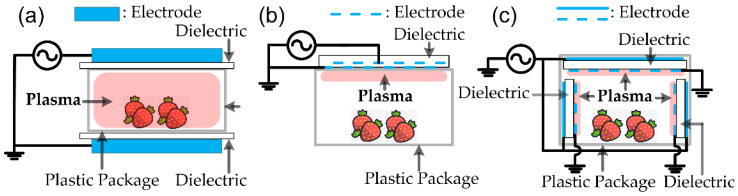
Schematic diagram of typical configurations for the generation of in-package cold plasma: (**a**) in-package plasma using planar-type DBD source located outside the package, (**b**) in-package plasma using surface-discharge type DBD source positioned outside the package, and (**c**) in-package plasma using surface-discharge type DBD source positioned inside the package. (Adapted with permission from Ref. [88]. 2019, Elsevier).

To create an in-package plasma system, the plasma source is typically integrated into the packaging material, as shown in Figure 7. The in-package plasma can be generated using planar-type DBD and surface-discharge type DBD. The plastic packaging materials for the in-package plasma are usually polymer-based materials such as LDPE (low density polyethylene), a PE pouch, a rigid PP/Cryovac pouch, and PET films. Without additional processing steps, this generates the plasma directly on the product’s surface. One of the advantages of in-package plasma is that it can be used to inactivate micro-organisms on products sensitive to heat or other processing methods. Rana et al. studied microbial reduction in strawberries using the in-package plasma [82]. The DBD system consisted of Al plate electrodes (gap: 30 mm), and the in-package plasma was generated inside the LDPE packet at a voltage of 60 kV (power frequency: 50 Hz). The LDPE was filled with air and contained the strawberry sample. Thus, the LDPE acted as both the container and the dielectric barrier. After plasma exposure, the reduction in bacteria, yeast, and mold was 2.1-log_10_ CFU/g. Here, CFU (colony-forming unit) is a unit, used to estimate the number of microbial cells. In addition, an n log_10_ reduction means that the concentration of remaining contaminants is only 10^−n^ times that of the original. For example, a 1-log_10_, 2-log_10_, 3-log_10,_ and 4-log_10_ corresponds to 90%, 99%, 99.9%, and 99.99% reduction, respectively, from the initial concentration. Mahonot et al. reported the reduction in the natural microflora of carrots after in-package plasma [85]. The plasma was generated using planar-type DBD (60 Hz, 80–100 kV), as shown in Figure 7a. The DBD system comprised two Al plate electrodes (diameter: 158 mm) and a polypropylene dielectric layer (thickness: 2 mm). A PP box containing carrots was sealed with polymeric film (Cryovac BB3050, Sealed Air Corp. Charlotte, NC, USA) and positioned between two electrodes. After the plasma treatment, a 2.1-log_10_ CFU/g reduction was observed for aerobic mesophiles and yeast. Zhao et al. investigated the reduction in yeast and micro-organisms on fermented vegetables (radish paocai) using the in-package plasma treatment. The DBD system comprised two Al circular electrodes and two PP barrier layers. The packed paocai was positioned between two barrier layers (gap: 40 mm) and was plasma treated three times at a voltage of 60 kV (power frequency: 60 Hz) for 60 s. After the treatment, the reduction rate of micro-organisms ranged from 4.54 to 5.61 log CFU/g. Los et al. and Ziuzina et al. also reported the reduction in bacteria, fungi, and pest insects using in-package plasma [97,98]. The DBD system consisted of two round Al electrodes (diameter: 15 mm) and a PP container with a sealed PP bag (Cryovac B2630, Sealed Air Corp., Charlotte, NC, USA). The wheat and pest insect (red flour beetle) were plasma-treated at 80 kV (power frequency: 50 Hz). The reduction rate for bacteria and fungi on wheat was 1.5 and 2.5 log10 CFU/g, respectively. A mortality of 95.0–100% for preadult stages can be achieved within seconds of treatment, but longer plasma exposure (5 min) is required to kill adult insects. In-package plasma can be used to sterilize fresh produce or other food products without damaging the product or altering its quality. In addition, in-package plasma can be used in various other applications, such as medical devices, electronics, and other products requiring surface sterilization or decontamination. Overall, in-package plasma is a promising technology that has the potential to improve the safety and quality of a wide range of products while also reducing the need for additional processing steps and increasing efficiency in various industries.

### 3.4. Comparison of CF-Derived Plasma and Cold Atmospheric Pressure Plasma Jet

**Figure 8:**
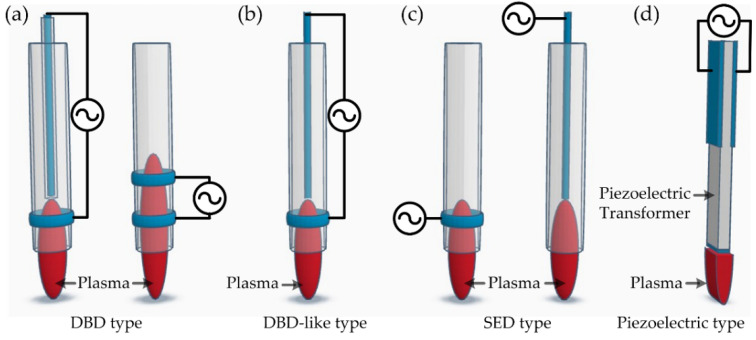
Schematic diagram of typical configurations for the generation of cold atmospheric pressure plasma jet: (**a**) dielectric barrier discharge (DBD) jets, (**b**) DBD-like discharge jet, (**c**) single electrode discharge (SED) jets, and (**d**) piezoelectric direct plasma jet.

Cold atmospheric-pressure plasma jets (APPJs) are a point of interest in many fields, and their applications range from lab-scale applications to industrial production [99,100,101,102,103]. Especially, compact, affordable, easy-to-use, and flexible plasma tools have been in great demand in lab-scale research. In fact, the cost is one of the important factors in lab-scale applications. There are two major cost factors in plasma generation equipment, which are the vacuum equipment and power source. Atmospheric plasmas do not require vacuum equipment, and APPJ satisfies these needs. In the CF-derived plasma, the power source is inexpensive, and it is possible to make it yourself because the structure of the power source is very simple. In addition, atmospheric pressure DBD discharge is possible, as mentioned in the previous session. In conclusion, APPJ and CF-derived plasma are cost-effective and easy to use, allowing a variety of applications. Figure 8 shows the schematic diagram of typical configurations for APPJ. The kINPen is a representation of a cold APPJ, which was developed at the Leibniz Institute for Plasma Science and Technology and commercialized by neoplas GmbH [104,105]. The schematic structure of kINPen is shown in Figure 8b. The kINPen is typically driven at a sinusoidal frequency of 1 MHz and can be operated with an inert gas and molecular gas mixture (up to 2%). The gas temperature is ~40 °C, the plasma temperature is up to 4 eV, and the estimated electron density is ~10^14^ cm^−3^ [104]. These properties are suitable for the processing of biomaterials and are applied to medicine, biology, dental treatment, genetics, etc. Also, this is used in the surface modification of many materials, surface cleaning, and decomposition of contaminants. The piezoelectric cold plasma generator (PCPG) is also another type of APPJ pencil [106], which is based on the resonant piezoelectric transformer [107]. Piezoelectric transformer, which is a simple PZT (PbZr_1−x_Ti_x_O_3_) ceramic rod, has a step-up ratio of about 1000 times and an output voltage of 10 kV or more is possible with a low input power of 25 W. PCPG was commercialized by relyon plasma GmbH, and the schematic diagram is shown in Figure 8d. PCPG can use various discharge modes, including DBD mode. This can operate with very low power input (typically ~10 W), and the advantage is the wireless plasma pencil. The kINPen and PCPG have the advantages of affordable equipment, a low gas temperature, the use of additive gas, and excellent portability. For CF-derived plasma, the power construction cost is very low. Especially in the case of atmospheric DBD discharge, plasma generation equipment can be inexpensively built since vacuum equipment is not required. In addition, the plasma volume can be easily increased to change the electrode configuration and reduce sample damages due to the production of slow-changing, discontinuous discharge. The comparison between kINPEN-, PCPG-, and CF-derived plasma is summarized in Table 4.

## 4. Conclusions

This review describes the characteristics and applications of plasma using a frequency of 50/60 Hz as the plasma power source. Due to low-frequency, the plasma reactor configurations in most plasma processes are of the CCP type. This method can be utilized in a wide range of applications, such as thin film deposition, surface oxidation/nitridation of thin films, and surface treatment of polymers. This method exhibits properties similar to those of DC-generated plasma but with electrically bipolar features, which prevent damage to the substrate due to charge accumulation. Furthermore, unlike DC power, this process can be applied to almost all materials, including dielectrics and polymers. The simple structure and low cost of CF power are particularly advantageous, making it a viable option for laboratory-scale manufacturing and use. Consequently, the CF-derived plasma process is helpful at a laboratory scale due to its simplicity, and the results for the plasma processes are also outstanding.

## Figures and Tables

**Table 1:** Summary of plasma parameters of different plasma generating methods.

Type	Frequency	Pressure (Torr)	*T_e_* (eV)	*n_e_* (cm^−3^)	Ref.
Positive Column	DC	10^−2^–10	1–3	~10^9^–10^11^	[2,3,4]
CCP	DC, CF 13.56 MHz	10^−2^–1	1–5	~10^8^–10^10^	[2,3,27,28,29,41]
ICP	13.56 MHz	10^−4^–10^−1^	1–10	10^11^–10^12^	[2,3,4,41]
Magnetron Sputtering	13.56 MHz	10^−3^–1	1–5	10^10^–10^12^	[2,3,4,7,41]
ECR	2.45 GHz	10^−4^–10^−2^	2–7	10^10^–10^12^	[2,3,4,41]
Microwave plasma (Surfatron)	2.45 GHz	760	5	10^12^–10^15^	[42]
Microwave plasma (SLAN)	2.45 GHz	760	5	10^11^	[42]
DBD	8 kHz 13.56 MHz	760	1–3	10^14^	[43,44]
Thermal Arc	30 A-30 kA	76–76,000	1–10	10^15^–10^19^	[1,2,3]
Non-complete Thermal Arc	1-30 A	10^−3^–100	0.2–2	10^14^–10^15^	[1,2,3]

**Table 2:** Comparisons of thermal and non-thermal plasma properties [41,49,50].

Properties	Thermal Plasma	Non-Thermal Plasma
Temperature	$T_{e}≳T_{i}\approx T_{n}$	$T_{e}\gg T_{i}\approx T_{n}$
Electron density (cm^−3^)	10^15^–10^20^	<10^13^
Heating	Joule heating Thermal heating	Electron impact process
Characteristics	High gas temperature Low excitation selectivity	Low gas temperature High excitation selectivity
Examples	Arc discharge	Glow discharge

**Table 3:** Comparisons of direct current (DC)-, commercial frequency (50/60 Hz)-, and radio frequency (RF, 13.56 MHz)-derived plasma characteristics in CCP-type discharge [2,3,27,28,29,41].

Properties	DC	CF	RF
Power frequency	0	50/60 Hz	13.56 MHz
Discharge type	Continuous	Discrete	Continuous
Main heating mechanism	Ohmic	Ohmic	Ohmic, Stochastic
Deposition/treatment material	Metal	Metal, dielectric	Metal, dielectric
Deposition/treatment rate	Lower in DC and RF discharge		
Plasma parameters	Similar in DC and RF discharge (*T_e_*: 1–5 eV, n_e_: ~10^8^–10^10^)		

**Table 4:** Comparisons between kINPen-, PCPG- (piezoelectric cold plasma generator), and CF-derived plasma as lab-scale equipment.

Type	Frequency	Pressure (Torr)	Characteristics	Ref.
kINPen	Typically, 1 MHz	~760	Low gas temperature: ~40 °C *T*_e_ = 4 eV, n_e_ = ~10^14^ cm^−3^ Wired portable Affordable cost	[95]
PCPG	40-90 kHz	~760	Low gas temperature: ~40 °C Low input power: typically, 10 W Wireless portable Affordable cost	[97]
CF-derived Plasma	50/60 Hz	~1	*T*_e_ = 1.4–3.2 eV, n_e_ = ~10^8^ cm^−3^ Inexpensive power source	[27,28,29,30,49]
		~760	Inexpensive power source High voltage: 50–80 kV	[89,90]

## Data Availability

Not applicable.
